# Applications of New Breeding Technologies for Potato Improvement

**DOI:** 10.3389/fpls.2018.00925

**Published:** 2018-06-29

**Authors:** Amir Hameed, Syed Shan-e-Ali Zaidi, Sara Shakir, Shahid Mansoor

**Affiliations:** ^1^Department of Bioinformatics and Biotechnology, Government College University, Faisalabad, Pakistan; ^2^Agricultural Biotechnology Division, National Institute for Biotechnology and Genetic Engineering, Faisalabad, Pakistan

**Keywords:** CRISPR, genome editing, nutritional quality, potato, TALEN

## Abstract

The first decade of genetic engineering primarily focused on quantitative crop improvement. With the advances in technology, the focus of agricultural biotechnology has shifted toward both quantitative and qualitative crop improvement, to deal with the challenges of food security and nutrition. Potato (*Solanum tuberosum* L.) is a solanaceous food crop having potential to feed the populating world. It can provide more carbohydrates, proteins, minerals, and vitamins per unit area of land as compared to other potential food crops, and is the major staple food in many developing countries. These aspects have driven the scientific attention to engineer potato for nutrition improvement, keeping the yield unaffected. Several studies have shown the improved nutritional value of potato tubers, for example by enhancing *Amaranth Albumin-*1 seed protein content, vitamin C content, β-carotene level, triacylglycerol, tuber methionine content, and amylose content, etc. Removal of anti-nutritional compounds like steroidal glycoalkaloids, acrylamide and food toxins is another research priority for scientists and breeders to improve potato tuber quality. Trait improvement using genetic engineering mostly involved the generation of transgenic products. The commercialization of these engineered products has been a challenge due to consumer preference and regulatory/ethical restrictions. In this context, new breeding technolgies like TALEN (transcription activator-like effector nucleases) and CRISPR/Cas9 (clustered regularly interspaced palindromic repeats/CRISPR-associated 9) have been employed to generate transgene-free products in a more precise, prompt and effective way. Moreover, the availability of potato genome sequence and efficient potato transformation systems have remarkably facilitated potato genetic engineering. Here we summarize the potato trait improvement and potential application of new breeding technologies (NBTs) to genetically improve the overall agronomic profile of potato.

## Introduction

The rising food demand in a populating world will require a proportional increase in the food source. In contrary, several factors like climatic change, industrialization, and urbanization have overburdened the existing agriculture lands and food resources (Badami and Ramankutty, 2015). Other factors causing food decline include various biotic and abiotic stresses continuously affecting crops worldwide. With the technological advancements and joint public-private partnership, several crops with enhanced nutritional profile have been developed using the existing gene pool (Ricroch and Henard-Damave, 2016; Ma X. et al., 2017).

Potato (*Solanum tuberosum* L.), a solanaceous food crop, is ranked fourth among the major staple crops after maize, rice, and wheat. It can provide more carbohydrates, proteins, minerals, and vitamins per unit area of land and time as compared to other potential food crops (Zaheer and Akhtar, 2016). In addition to being a raw marketable product, potato is largely used in industry for making processed food products, alcohol, starch, animal feed and for biofuel production (Scott and Suarez, 2012; Liang and McDonald, 2014). Short crop duration and wide climatic adaptability have facilitated potato to spread across diverse geographical borders from its South American origin. Today, more than three thousand potato cultivars are widely distributed in more than 125 countries, particularly under temperate, subtropical and tropical regions covering a major economic share in the global agricultural market (Birch et al., 2012). For the last two decades, potato cultivation and utilization have also been notably increased in developing countries such as Bangladesh, India, and China (Zaheer and Akhtar, 2016).

In terms of nutrition, potato is a complex source of nutrients (vitamins, carotenoids, anti-oxidant phenolics, proteins, magnesium etc.), and some anti-nutrients (primarily glycoalkaloids). On average, potato tubers contain 77% water, 20% carbohydrates, and less than 3% of proteins, dietary fiber, minerals, vitamins and other compounds (Zaheer and Akhtar, 2016). Comprehensive information regarding the tuber composition of different potato cultivars is described by Burlingame et al. (2009). In low-income food-deficit countries (http://www.fao.org/countryprofiles/lifdc/en/), potato could replace other high-priced foods and can be sustainably used as a cheap food giving enough calories (93 kcal/100 g tuber) to sustain a normal life (Burlingame et al., 2009). The global importance of potato is unquestionable and to commercialize its role in defeating food-shortage, poverty, and predominantly malnutrition, United Nations celebrated 2008 as the “International Year of the Potato” (http://www.fao.org/potato-2008/en/).

Several breeding and molecular approaches have been employed for trait improvement in potato. Conventional breeding techniques for potato improvement are directed to increase yield, processing, and storage-quality (Halterman et al., 2016). Potato breeders incorporated resistance against early and late blight disease by crossing hybrid lines with wild species (*S. brevidens* and *S. bulbocastanum*) which inherited resistance against fungal pathogens (Naess et al., 2000; Tek et al., 2004). Although conventional breeding has been successfully employed for targeted trait improvement with less intraspecific variability, the progress is relatively slow and limited due to the phenotypic characterization of leading individuals in successive generations. In addition, the search of useful genetic variability in wild relatives could be laborious and its introgression in cultivated variety can be another challenging task. High heterozygosity and tetraploid nature of the potato genome (Consortium, 2011) are major drawbacks in breeding efforts to improve potato because of allelic suppression at each breeding cross (Lindhout et al., 2011). Other factors may include intra-species incompatibilities and inbreeding depression that causes failure in trait incorporations in polyploid crops through conventional breeding.

In this context, new breeding technologies (NBTs) offer a leading hand for trait improvement in crop plants and provide a platform for precise and robust plant genome editing. These NBTs include, but are not limited to, the cutting-edge genome editing approaches like clustered regularly interspaced short palindromic repeats/CRISPR associated 9 (CRISPR/Cas9), transcription activator-like effector nucleases (TALENs) and zinc-finger nucleases (ZFNs) (Jinek et al., 2012; Schaart et al., 2016; Weeks et al., 2016). Although developed recently, CRISPR system has been effectively employed for trait improvement of several economically important crops like wheat, maize, rice, cassava, cotton, soybean, and potato (Puchta, 2017). The introduced traits include herbicide tolerance, fungal/bacterial/viral disease resistance, drought tolerance, and increased shelf life, leading to overall improved quality and production. The working methodology and the anticipated role of these NBTs in plant genetic engineering have been extensively reviewed (Bortesi and Fischer, 2015; Mahfouz et al., 2016; Schiml and Puchta, 2016; Puchta, 2017; Weeks, 2017; Zaidi et al., 2017a,b, 2018). The current review provides a comprehensive information on different genetic approaches, including NBTs, that have been successfully employed to enhance the nutritional value of potato (Tables 1, 2). Moreover, we summarize the data on transgenic potato commercialized so far (Table 3) and the major concerns associated with their regulatory approvals.

**Table 1:** Transgenic potato enhanced for nutritional traits.

**Nutrient**	**Cultivar(s)**	**Targeted gene(s)**	**Molecular function**	**Technology used**	**Results**	**References**
Protein	*S. tuberosum* var-A16 and some other local cultivars	*Amaranthus hypochondriacus*1 (*AmA1*)	Encodes seed-specific albumin protein	Transgenesis	Up to 60% increase in tuber protein	Chakraborty et al., 2000, 2010
	Desirée, Breeding lines (MGL6 and MGL34)	*A. thaliana* cystathionine γ-synthase (At*CGS*); *S. tuberosum* methionine γ-lyase (St*MGL*)	At*CGS*: methionine biosynthesis; St*MGL*: prevent methionine degradation	Transgenesis and endogenous gene silencing	Up to 2-fold increase in tuber methionine	Kumar and Jander, 2017
Vitamins	Ranger Russet	*A. thaliana*_L_-galactose phosphorylase (*GDP*)	Involved in ascorbate biosynthesis pathways	Transgenesis	Up to 3-fold increase in tuber vitamin-C	Bulley et al., 2012
	Desirée	Cauliflower Orange (*Or*)	Regulate carotenoid accumulation	Transgenesis	Up to10-fold increase in tuber pro-vitamin A	Li et al., 2012
	Taedong Valley	Rat-cells _L_-gulono-γ-lactone oxidase (*GLOase*)	Involved in vitamin-C biosynthesis pathways	Transgenesis	Enhanced (141%) _L_-Ascorbic acid in tubers	Upadhyaya et al., 2010
Carotenoid	Desirée, Breeding line (91E22), Yema de Huevo	beta-carotene hydroxylase (*bch*)	Involved in carotenoid biosynthesis pathways	RNAi silencing	Significant increase in tuber β-carotene and lutein	Van Eck et al., 2007
	Desirée	Three bacterial (*Erwinia*) genes (*CrtB, CrtI*, and *CrtY*)	Involved in β-carotene biosynthesis pathways	Transgenesis	Up to 20-fold increase in tuber carotenoids	Diretto et al., 2007
Calcium	Russet Norkotah	*A. thaliana* H^+^/Ca^2+^ transporter (*sCAX1*)	Involve in H^+^/Ca^2+^ transport	Transgenesis	Up to 3-fold increase in tuber Ca	Park et al., 2005
Caffeoyl quinic acids (CQAs)	Desirée	*A. thaliana* flavonol- specific transcriptional activator *(*At*MYB12)*	Activates the CQA biosynthetic pathway	Transgenesis	More than 3-fold increase in tuber CQAs	Li et al., 2016
Starch	Kuras	Starch branching enzyme family (*SBEI and SBEII*)	Involved in starch branching pathways	RNAi silencing	Significant increase in tuber amylose	Andersson et al., 2006
Triacylglycerol (TAG)	Atlantic	*WRI1, DGAT1, OLEOSIN*	Involve in fatty acid biosynthesis and TAG assembly	Transgenesis	Up to 100-fold increase in tuber TAG	Liu et al., 2017
**ANTI-NUTRIENT**						
Steroidal glycoalkaloids (SGAs)	Desirée	GLYCOALKALOID METABOLISM 4 (*GAME4*)	Involved in SGAs biosynthesis pathways	RNAi silencing	Up to 74-fold decrease in tuber SGAs	Itkin et al., 2013
Acrylamide	Katahdin, Russet Burbank	Vacuolar acid invertase (*VInv*); asparagine synthetase genes (St*AS1* and St*AS2*)	Involved in accumulation of reducing sugars inside vacuole and acrylamide precursors	RNAi silencing	Significant reduction in acrylamide content	Bhaskar et al., 2010; Zhu et al., 2016

*Transgenesis: Introducing an exogenous gene “transgene” into a living organism so that the organism will stably exhibit a new property and transmit that property to next generation*.

**Table 2:** Applications of some new breeding technologies for potato trait improvement.

**Objective**	**Target gene(s)**	**Molecular function**	**Edit tool**	**Results**	**References**
Reduced anti-nutrient element	Sterol side chain reductase 2 (*SSR2*)	Involved in cholesterol biosynthesis	TALENs	Reduced level of toxic steroidal glycoalkaloids	Sawai et al., 2014
Targeted mutagenesis	Aux/IAA gene family (St*IAA2*)	Involved in Auxin/indole-3-acetid acid proteins synthesis	CRISPR/Cas9	Altered Aux/IAA protein expression	Wang et al., 2015
Targeted mutagenesis	Acetolactate synthase gene (*ALS*)	Biosynthesis of branched-chain amino acids	TALENs	Site-specific mutation with frequency rate (7–8%)	Nicolia et al., 2015
Targeted mutagenesis	Acetolactate synthase1 gene (St*ALS1*)	Biosynthesis of branched-chain amino acids	CRISPR/Cas9	Site-specific mutation	Butler et al., 2015
Altered starch composition	Granule-bound starch synthase (*GBBS*)	Involved in starch synthesis pathway	TALENs	Targeted mutation	Kusano et al., 2016
Reduced anti-nutrient element	Vacuolar invertase gene (*VInv*)	Accumulation of reducing sugars inside tubers	TALENs	Reduced acrylamide and resistance against CIS	Clasen et al., 2016
Targeted mutation	Acetolactate synthase1 gene (*ALS1*)	Biosynthesis of branched-chain amino acids	Geminivirus replicon (GVR)-mediated TALENs and CRISPR/Cas9 delivery	Site-specific mutation and herbicide susceptibility	Butler et al., 2016
Altered starch quality	Granule-bound starch synthase (*GBBS*)	Involved in starch synthesis pathway	CRISPR/Cas9	High mutation frequencies at targeted sites (up to 67%)	Andersson et al., 2017
Targeted mutations	4-alpha-glucan branching enzyme (*SBE1*), Vacuolar invertase 2 gene (St*vacINV2*)	*SBE1*: Involved in starch synthesis pathways; *StvacINV2*: Accumulation of reducing sugars inside tubers	TALENs	Induced mutations at targeted sites	Ma J. et al., 2017

**Table 3:** Engineered potato commercialized so far or in regulatory pipeline.

**Edited trait**	**Product**	**Status**	**Manipulated gene(s)**	**Technology used**	**Developer**	**Referencess**
Insect resistance	NewLeaf^TM^	Approved in 1995 and withdrawn in 2001	Bacterial *Cry3A* gene to provide resistance to CPB	Transgenesis	Monsanto®	Thornton, 2003
Insect and viral resistance	NewLeaf Plus^TM^	Developed in 1998 and withdrawn in 2001	Bacterial *Cry3A* gene and viral replicase gene to provide resistance to CPB and PLRV	Transgenesis	Monsanto®	Lawson et al., 2001
Improved processing quality	Innate^TM^ 1.0	Limited commercial launch in May 2015	Potato *VInv* and *Asn1* gene to provide resistance to CIS and acrylamide formation	RNAi	J.R Simplot®	Waltz, 2015
Improved processing quality and Late Blight resistance	Innate^TM^ 2.0	Approved in 2017 by EPA and FDA	Potato *VInv, Asn1* and *Rpi-vnt1* gene for multiple trait introgression	TALENs	J.R Simplot®	Halterman et al., 2016
High starch content	Amflora^TM^	March, 2010 approved by the EC	Potato *GBSS* gene to control amylose synthesis	RNAi	BASF Plant Science	Halterman et al., 2016
Insect resistant	Elizaveta Plus and Lugovskoi Plus	May, 2015 Approved for cultivation in Russia	Bacterial Cry3A gene to provide resistance to CPB	Transgenesis	Russian Academy of Sciences	Korobko et al., 2016
Blight resistance	*	Confined experimental trails, EU	*Rpi-vnt1* gene transfer from wild potato to provide resistance to Late Blight	Cisgenesis	BBSRC, UK	Ricroch and Henard-Damave, 2016
Blight resistance	*	Field trials, EU	Introgression of some *R*-genes from wild potato cultivars to provide resistance to Late Blight	Cisgenesis	Plant Research International, WUR, Netherlands	Haverkort et al., 2009
Insect resistance	SpuntaG2	Field trials, South Africa, USA	Bacterial *Cry2a1* gene for resistance to potato tuber moth	Transgenesis	Collaborative project under USAID	Douches et al., 2010
Increased Protein	*	R&D	Introgression of *AmA1* gene to enhance tuber protein content	Transgenesis	NIPGR, India	Chakraborty et al., 2010
Vitamin-C biofortified	*	R&D	Introgression of *GDP* gene to enhance tuber ascorbate content	Transgenesis	J.R Simplot® and University of Auckland, New Zealand	Bulley et al.

* *Unknown. R&D, Research and Development; EPA, U.S. Environmental Protection Agency; FDA, Food and Drug Administration; EC, European Commission; EU, Europe; CPB, Colorado Potato Beetle; PLRV, Potato leafroll virus; Cry3A and Cry2a1, Bacillus thuringiensis genes; VInv, Vacuolar acid invertase; Asn1, Asparagine synthetase-1 gene; Rpi-vnt1, Late Blight resistance gene from wild potato (Solanum venturii); AmAI, Amaranthus hypochondriacus1; GDP, Arabidopsis thaliana _L_-galactose phosphorylase; GBBS, Granule bound starch synthase; BBSRC, Biotechnology and Biological Sciences Research Council; WUR, Wageningen University and Research Centre; NIPGR, National Institute of Plant Genome Research*.

## Constrains to potato productivity and quality

The sustainable potato production faces a number of challenges due to biotic stresses (viruses, bacteria, fungal, insect pests) and abiotic stresses (drought, salinity, temperature, frost and post-harvest problems, i.e., accumulation of reducing sugars during cold storage).

### Diseases and insect pests affecting potato

Most of the potato diseases are due to the diverse prevalence of phytopathogens of which viruses are of prime importance. Cultivated potato is susceptible to around 40 different viral and 2 viroid species (Salazar, 1996). Among the dominating viruses, *Potato virus Y* (PVY, genus; potyvirus), *Potato leafroll virus* (PLRV, genus; polerovirus), and *Potato virus X* (PVX, genus; potexvirus) are probably the most diverse and devastating viruses infecting potato worldwide (Fletcher, 2012; Hameed et al., 2014; Steinger et al., 2014). Viral diseases appear as necrotic strains on leaves/tubers, mosaic, and overall stunted growth to plant, leading to reduced yield and poor-quality tubers. Moreover, several bacterial diseases (soft rot/blackleg caused by *Dickeya solani*, common scab caused by *Streptomyces scabies*) (Buttimer et al., 2017), and fungal diseases (late blight caused by *Phytophthora infestans*, powdery scab caused by *Spongospora subterranea*; Arora et al., 2014; Balendres et al., 2016) are also severely deteriorating potato quality worldwide. Late blight affected potato plants exhibit water-soaked leaves having necrotic lesions and irregular colored tissue in tubers making them hard, dry and more susceptible to other microbial diseases.

Virus resistance in potato has been engineered through different approaches ranging from simple plant breeding to advanced genetic engineering. Transgenic approaches to engineer virus resistance in potato are seemed to be more appropriate than conventional breeding due to its polyploid nature making difficulties for the introgression of resistance genes. Thus, RNA interference (RNAi)- mediated resistance targeting viral coat protein (CP) region has been demonstrated in potato, where single or multiple RNA viruses have been targeted with different success levels; such as PVY- resistance (Missiou et al., 2004); PVY, and PLRV-resistance (Chung et al., 2013); and PVX, PVY, and *Potato virus S* (PVS)-resistance (Hameed et al., 2017). The current scenario of GM potatoes being commercialized in some countries encompasses viral resistant potatoes generated through genetic engineering (Mathur et al., 2017).

Potato pests cause direct damage to potato crop in the forms of necrosis, deformations of plant tissues and/or indirect damage by facilitating the pathogen dispersal, especially for viruses. Important destructive insects affecting potato include Colorado potato beetle (*Leptinotarsa decemlineata*) (Casagrande, 2014), peach-potato aphid (*Myzus persicae*) (Bass et al., 2014), potato tuber moth (*Phthorimaea operculella*) (Liu et al., 2018) etc. The extensive use of chemical insecticides on insect pests has led to the evolution of insecticide-resistance in particular insects, thus posing alarming threats. To effectively control their incidence *in planta*, genetic engineering has offered some promising solutions like introgression of insecticidal proteins/toxins (Palma et al., 2014), RNAi-mediated insect resistance (Zhang J. et al., 2017) and CRISPR-Cas9-mediated crop protection (Douglas, 2017) etc. In potato, RNAi technology was used to engineer resistance against Colorado potato beetle (Zhang et al., 2015). Sap transmitted RNAi reagents (long double-stranded (ds) RNAs in chloroplasts) resulted in more than 80% of the reduced expression of insect targeted genes (β-actin gene) and triggered a lethal RNAi response destructive to its larvae (Zhang et al., 2015).

### Agronomic attributes affecting potato

Farming systems comprising of different agronomic attributes like tillage, nutrient management, and crop rotation significantly affect potato tuber productivity and quality. Due to its shallow root-system, potato needs a fair supply of nutrient inputs to maintain its tuber vigor and yield (Alva et al., 2011). Research has shown the influence of different farming practices on tuber quality parameters like tuber dry mass accumulation, enhanced nutrient/mineral concentration and yield improvements (Brazinskiene et al., 2014; Tein et al., 2014; Nyiraneza et al., 2015). Through adopting a potato-legume crop rotation, (Qin et al., 2017) observed a positive influence on soil microbiota coupled with significantly improved tuber yield up to 19% when compared with the continuous cultivation of potato crop only. Integrated crop rotations with an exogenous supply of organic and mineral [nitrogen (N), phosphorous (P), and potassium (K)] fertilizers significantly influenced potato tuber N, nitrate, magnesium (Mg) and P concentrations when compared with non-fertilized controls (Tein et al., 2014). Leonel et al. (2017) analyzed five potato cultivars for their tuber chemical composition in response to different concentrations of available P supplemented with uniform cultural practices. Potato tubers fertilized with increased P exhibited a significant positive influence of tuber dry matter and protein/starch contents and a lower concentration of total sugar contents (Leonel et al., 2017). The chemical composition of potato tubers is a prerequisite for determining the nutritional and processing quality of industrial perspectives. Understanding the importance of organic products, Lombardo et al. (2017) evaluated the nutritional value of organic vs. conventionally grown potatoes. Field trials of yellow-fleshed potato cultivars growing under organic cultivation produced high-quality tubers having enhanced concentrations of phenolics, reduced nitrate and a more attractive tuber flesh color (Leonel et al., 2017).

### Climatic and soil factors affecting potator

A number of abiotic stresses ranging from soil to climate significantly affect the potato productivity and quality during its growth and/or after harvest. Potato cultivation performs better under cool condition (19°C) and is vulnerable to high temperatures (Kim et al., 2017). A fairly low temperature promotes the first tuber set and sudden elevations in temperature during this early tuberization significantly affect tuber yield and size (Zhou et al., 2017). A drastic reduce in potato tuber yield ranging from 3 to 11% per 1°C rise in temperature was observed across various geographical locations (Fleisher et al., 2017; Kim et al., 2017). During the early growth stage of potato, low freezing and/or frost attacks severely damage the young plantlets and could reduce the tuber yield and quality (Chang et al., 2014).

Drought and salinity are other important abiotic stresses having adverse effects on potato cultivation. Potato cultivars respond differentially to drought conditions and mostly exhibit various physiological and morphological changes in tuberization and plant growth (Chang et al., 2018). The major impact of this water stress has been recorded in the form of reduced tuber yield due to a loss of internal water pressure during tuber bulking and maturation (Stark et al., 2013). Salinity, another acute abiotic stress causes many inhibitory effects on plant growth and development (Parihar et al., 2015). Salinity stress in potato severally affects its productivity by causing enhanced oxidative stress, reduced photosynthesis and significantly reduced tuber yield. Research efforts through interspecific breeding in potato resulted in improved tolerance to salinity and oxidative stresses (Jbir-Koubaa et al., 2015).

### Post-harvest factors affecting potato

The post-harvest storage of potato tubers is another criterion for determining their end-product processing quality. Usually, this long-term storage is accompanied with various storage diseases like soft rot, black dot, and *Fusarium* dry rot which significantly reduces the tuber quality and unfit it for further processing (Usall et al., 2016). Soil-transmitted black dot, caused by *Colletotrichum coccodes* imparts brown necrotic lesions/stains on tuber skin and promotes their rapid decay (Brierley et al., 2015). Storage temperature and durations are two important factors determining the tuber susceptibility to black dot disease and could be managed to prevent the quality losses in potato (Peters et al., 2016). The tuber harvesting date also influences tuber quality during the long-term storage. Makani et al. (2015) observed the storage quality of potato cultivars in response to harvest time and subsequent storage. The results showed that the tubers harvested at full maturity retained their quality during storage in contrary to early harvested tubers (less mature), which exhibited a significant loss in tuber dry matter and ascorbic acid contents (Makani et al., 2015). Potato dormancy characteristics are other challenging factor determining the tuber quality during storage. Dormancy is an innate ability to sustain sprouting for a time and after its natural breakage, sprouting starts which cause various quality issues. Dormancy could be regulated in potato tubers through the topical applications of phytohormones, such as ethylene which has the ability to suppress bud formation/sprouting (Sonnewald and Sonnewald, 2014).

Potatoes are usually subjected to cold-storage (4–8°C) in order to ensure a continuous supply to consumers/markets throughout the year. This cold-storage is accompanied by elevated levels of reducing sugars in the tuber, a phenomenon termed as “Cold-Induced Sweetening: CIS” (Bhaskar et al., 2010). During CIS, tuber starch content is biochemically converted to sugars (sucrose) through the cohesive activity of several hydrolytic enzymes (Sowokinos, 2001). The elevated sucrose is subsequently transported inside a vacuole where it is further reduced to glucose and fructose through the activity of a host gene (vacuolar acid invertase, *VInv*) (Sowokinos, 2001; Bhaskar et al., 2010). The CIS affected tubers when used as feedstock for high-temperature processing gives rise to the accumulation of a dark brown, bitter tasting product, i.e., acrylamide. The rising acrylamide contents in food products is a huge concern to global food safety as well as to end-chain consumers (Vinci et al., 2012). Several reports depict the alarming levels (up to 70%) of acrylamide in food products that mainly come through the intake of fries, chips and other fried potato products (Pedreschi et al., 2014; McCombie et al., 2016; Esposito et al., 2017).

## Enhancing nutrient contents in potato

For the last two decades, several efforts have been conducted to improve the nutritional traits of potato. The following section describes the information regarding nutrient enhancement in potato and is summarized in Table 1.

### Increased protein content

The risk of protein deficiency is more in the countries where people take protein-deficit diet as a staple food (Chakraborty et al., 2010). Unfortunately, the cultivated potato contains fewer proteins (0.85–4.2%) lacking lysine, tyrosine, and some other essential amino acids (Burlingame et al., 2009). To deal with this limitation, scientists have engineered potato with enhanced protein content through constitutive expression of tuber-specific gene, *Amaranthus hypochondriacus*1 (*AmA1*) (Chakraborty et al., 2000, 2010). The *AmA1* gene encodes for a seed protein, albumin: a non-allergic protein containing essential amino acids and considered safe for human/animal consumption [safety accredited by the World Health Organization (WHO)]. In transgenic potato, the enhanced protein (albumin) localizes inside cytoplasm/vacuole. The tubers of seven engineered potato cultivars showed an increased protein content up to 60% as compared to controls (Chakraborty et al., 2010). In addition to increased protein content, the transgenic potato also showed an accelerated rate of photosynthesis that ultimately increased the total biomass/yield of plants. Recently, methionine content (an essential amino acid involved in multiple cellular pathways) was significantly increased in transgenic potato cultivar (cv.) Desirée (Kumar and Jander, 2017). By using RNAi technology, overexpression of an exogenous gene *Arabidopsis thaliana* cystathionine γ-synthase (*AtCGS*), along with the suppression of a host gene *S. tuberosum* methionine γ-lyase (*StMGL*), resulted in nearly a double concentration of free methionine inside transgenic tubers as compared to control tubers (Kumar and Jander, 2017). Moreover, the experimental studies of engineered plants showed no morphological and yield differences when compared with control plants. Other studies were also conducted to increase the protein content in potato but met with limited success and yield penalties (Zeh et al., 2001; Dancs et al., 2008; Rinder et al., 2008; Galili and Amir, 2013).

### Increased vitamin and carotenoid contents

Several studies have been made to increase the vitamin content in potato, for example, expressing an exogenous gene, *A. thaliana*_L_-*galactose phosphorylase* (*GDP*) showed a 3-fold increase in ascorbate contents (vitamin C) (Bulley et al., 2012). Carotenoids are phytonutritive, anti-oxidative, lipophilic compounds (precursors to vitamin) present in many fruits and vegetables (Dellapenna and Pogson, 2006), and provide nutritional benefits in terms of increased vitamin uptake. Introduction of *cauliflower Orange* (*Or*) gene has shown a net increase in carotenoid content (pro-vitamin A) in cold-stored tubers (Li et al., 2012). Among other carotenoids (Lutein, zeaxanthin, violaxanthin, neoxanthin), β-carotene concentration is considerably low in potato (Ezekiel et al., 2013). RNAi approach was utilized to silence the β*-carotene hydroxylase* (*bch*) gene that showed a significant increase in β-carotene and lutein contents in the tubers (Van Eck et al., 2007). Another study reported a 20-fold increase in tuber carotenoid contents through expressing three bacterial genes involved in carotenoid biosynthesis (Diretto et al., 2007). Similarly, transgenic potato cv. Taedong Valley was produced, over-expressing *GLOase* gene (_L_-gulono-γ-lactone oxidase from rat cells) that showed an enhanced (141%) content of _L_-Ascorbic acid (vitamin C) (Upadhyaya et al., 2010).

### Increased calcium content

Being nutritious with several other elements, the cultivated potato is a poor source of Ca (Weaver et al., 1999). To address this deficiency, Park et al. (2005) utilized a transgenic approach through expressing an exogenous gene, *Arabidopsis H*^+^*/Ca*^2+^ *transporter* (*sCAX1*) in potato cv. Russet Norkotah. The regenerated plants expressing *sCAX1* gene showed a significant increase (up to 3-fold) of Ca contents in tuber as compared to controls. Field trials and morphological data from three consecutive crop generations proved the stable integration of enhanced Ca trait with no alteration in tuber yield and other growth/morphological characters. Potato with enhanced Ca contents could be potentially used as a dietary source, more specifically in countries where potato is a staple food (Park et al., 2005).

### Increased phenolic contents

In potato, 80% of the phenolic compounds are present in the form of caffeoyl quinic acids (CQAs) (Brown, 2005). Recently, Li et al. (2016) conducted a study to increase the CQAs content in potato tubers. Tuber specific constitutive expression of an exogenous gene, flavonol-specific transcriptional activator (*AtMYB12*: derived from *A. thaliana*) showed a significant increase (>3-folds) of CQAs and total flavonoid content. Importantly, they utilized a selectable marker-free approach to facilitate the downstream regulatory approvals (Daniell, 2002). The increased phenolic contents being imposing health benefits also induce some antimicrobial properties to plants, particularly with reduced fungal infections (Li et al., 2016).

### Increased starch contents

Potato tubers are a rich source of dietary starch and can provide a significant calorie intake in food-deficit countries. Starch is primarily composed of two structural components, amylose, and amylopectin, which are biosynthesized through cohesive actions of several enzymes. Extensive studies have been conducted to improve the digestible amylose content in potato by engineering different steps of starch biosynthesis pathway (Schwall et al., 2000; Hofvander et al., 2004). Tuber specific RNA silencing of two host genes (*SBEI, SBEII*), involved in starch branching pathway, resulted in the generation of potato with enhanced amylose content (Andersson et al., 2006). Recently, amylose contents were significantly increased (28–59%) in non-genetically modified potatoes by introducing a recessive allele (gene marker: *IAm*) from wild potato (*S. sandemanii*) into cultivated potato (*S. tuberosum*) through marker-assisted crossing (Krunic et al., 2018).

Plant-based oils are promising for the near future as a potential feedstock for a renewable energy. Currently, biofuel research is more focused on engineering crops with enhanced oil contents through genetic manipulation in lipids/triacylglycerol (TAG) synthesis pathways (Vigeolas et al., 2007; Vanhercke et al., 2014; Zale et al., 2016). Contrary to high starch content, oil (lipids) concentration is very low in potato tubers. Some recent studies have shown the enhanced TAG content in the engineered potato tubers (Hofvander et al., 2016; Liu et al., 2017). Tissue-specific constitutive expression of three genes (*WRI1, DGAT1*, and *OLEOSIN*) resulted in a 100-fold increase in TAG content in tuber as compared to controls (Liu et al., 2017). However, this TAG increase was also accompanied by a depletive nutritional effect in terms of significantly reduced starch (amylose) and accumulated sugar (sucrose) levels. Further exploration of this mechanism revealed a better understanding of negative impacts of TAG accumulation on tuber amylose and phosphate contents, as well as needs to optimize genetic engineering for particular traits (Mitchell et al., 2017).

## Reduction of anti-nutrient contents in potato

Another strategy to improve the nutritional quality of food is by reducing the anti-nutrient elements. There is no lethal toxicity reported with the consumption of potato as food (Zaheer and Akhtar, 2016), however, some anti-nutrient elements like steroidal glycoalkaloids (SGAs) (0.071–175 mg/100 g), primarily α-solanine, and α-choconine accumulate in tubers during crop maturation (Burlingame et al., 2009). These SGAs when present in higher amounts in food may cause neuro-toxic and/or nutrient absorption problems (Itkin et al., 2013). Biosynthesis of SGAs involves a concurrent expression of two key enzymes, uridine 5'-diphosphate, and glycosyltransferase which biochemically react with cholesterol, sugars, and other nitrogenous compounds to build up the glycoalkaloid molecules (Itkin et al., 2011). RNAi-mediated silencing of the host gene, *Glycoalkaloid metabolism 4* (*GAME4*) in potato showed a significant decrease (up to 74-fold) in SGAs content in leaves and tubers (Itkin et al., 2013). Importantly, many wild species of potato produce high levels of SGAs naturally (Gregory et al., 1981), therefore breeders must be careful to map the SGAs-gene (s) linkage with the desired traits when using the wild germplasm as a genetic resource.

Usually, raw potato is processed (fried, baked, mashed, microwaved) into various food products like snaps, fries, chips, *etc*. prior to eating (Zaheer and Akhtar, 2016). Sometimes, potato processing results in hazardous compounds causing obesity, cardiovascular diseases, and/or neurotoxicity. The first report of acrylamide, a potential neurotoxin and carcinogenic element, presence in potato fried products raised a debate among food regulatory authorities and processing industry (Tareke et al., 2002). Since then, several studies were conducted to explore the acrylamide formation during “Maillard Reaction,” a reaction among tuber asparagine contents, reducing sugars (primarily glucose and fructose) and free α-radicals present in cooking oil during high-temperature processing of potato (Stadler et al., 2002; Friedman, 2003; Vinci et al., 2012).

Contrary to cultivated tetraploid potato (*S. tuberosum*), some diploid species of wild potato are naturally resistant to CIS. Through quantitative trait loci (QTL) mapping and other molecular studies, scientists have identified some recessive genes associated with CIS resistance in wild species. Potato breeders tried to incorporate these genes in cultivated potato in order to reduce the CIS effect but met with limited success. Therefore, different molecular strategies have been applied to reduce the formation of acrylamide through indirectly mediating the CIS mechanism at the cellular level (Bhaskar et al., 2010; Li et al., 2013; Zhu et al., 2016; Hameed et al., 2018). Tuber-specific constitutive expression of *VInv* gene in anti-sense binary constructs resulted in significant reduction of reducing sugar content in cold-stored tubers. High-temperature processing of food products derived from these transgenic lines showed an 8-fold decrease in acrylamide content as compared to controls (Ye et al., 2010). In another study, RNAi-mediated simultaneous silencing of potato asparagine synthetase genes (*StAS1* and *StAS2*) and *VInv* gene significantly reduced the CIS process as well as asparagine content in transgenic potato cv. Russet Burbank (Zhu et al., 2016). Tubers derived from these CIS resistant transgenic lines showed a significant reduction (15-fold) of acrylamide content in fried potato products. The first-generation biotech potato (Simplot's Innate TM) was engineered to have lower reducing sugars levels and reduced asparagine contents to address the acrylamide forming problems during potato processing (Halterman et al., 2016).

## New breeding technologies used for increasing nutritional quality of potato

Gene pyramiding in polyploid crops using conventional breeding is a difficult, laborious, and time-consuming (Weeks, 2017). In potato, several breeding efforts have been made for particular trait improvement using wild species germplasm but met with limited success (Carputo and Barone, 2005). The presence of four copies (alleles) of genes in the tetraploid (2n = 4x = 48) genome of cultivated potato (*S. tuberosum*) makes it difficult for researchers/breeders to precisely edit the genome using conventional breeding tools (Consortium, 2011). Thus, NBTs such as CRISPR/Cas9, TALENs, and ZFNs offer great potential for expediting genome editing in a more precise and time-saving way (Mahfouz et al., 2016; Petolino et al., 2016; Schiml and Puchta, 2016).

In case of potato, both TALENs (Sawai et al., 2014; Nicolia et al., 2015; Clasen et al., 2016), and CRISPR/Cas9 (Butler et al., 2015; Wang et al., 2015) technologies have been efficiently utilized for precise genome editing (Table 2). In 2014, the first attempt of utilizing TALENs technology in potato genome editing paved the way for the next technology shifts. Sawai et al. (2014) utilized TALENs approach in potato (*S. tuberosum* cv. Sassy) to silence a host gene, *Sterol side chain reductase 2* (St*SSR2*) that is predominantly involved in cholesterol biosynthesis and a precursor to many toxic SGAs formations. Transgenic expression of TALENs constructs generated a site-specific mutation of variable size (nucleotide deletion/insertion) in four alleles of St*SSR2* gene. The transgenic potato with knock-out St*SSR2* activity showed a significant reduction of SGAs contents without affecting plant growth, thus eliminating an anti-nutritional factor (Sawai et al., 2014). In another study, protoplast delivery of TALENs constructs resulted in a significant mutation frequency (7–8%) at targeted gene loci, i.e., Acetolactate *synthase* (*ALS*) in transgenic potato cv. Desirée (Nicolia et al., 2015). Sequencing analysis of *ALS*-mutated lines confirmed the targeted protein disruption either through amino acid substitutions, truncations, and/or frameshift mutations and importantly mutated lines showed no phenotypic differences compared to controls (Nicolia et al., 2015). Wang et al. (2015) reported the use of CRISPR/Cas9 system for inducing efficient targeted mutagenesis in potato. *Agrobacterium*-mediated transformation of cells with CRISPR constructs resulted in efficient site-specific mutation in host gene, *Auxin/indole-3-acetic acid* (St*IAA2*) engineered for altered Aux/IAA protein expression (Wang et al., 2015). In a later study, Butler et al. (2015) targeted *Acetolactate synthase1* (*ALS1*) gene in potato through the *Agrobacterium*-mediated delivery of CRISPR/Cas reagents. Importantly, they utilized both tetraploid (*S. tuberosum* cv. Desirée) and diploid (MSX914-10-X914-10) potato as explant for their experiments. Stable expression of CRISPR/Cas reagents resulted in site-specific mutations (ranged from 3 to 60%) in *ALS* alleles and were stably heritable (87–100%) in successive diploid and tetraploid potato generations (Butler et al., 2015).

To improve the nutritional value of potato tubers, TALENs technology has been used to interrupt the *VInv* activity in order to reduce the accumulation of reducing sugars during cold-induced storage (CIS) (Clasen et al., 2016). Protoplast-mediated transformation of potato cv. Ranger Russet with TALENs constructs resulted in knockout of *VInv* alleles in transformed plants (Clasen et al., 2016). Interestingly, 5 out of 18 transformed lines showed a nearly complete silencing of *VInv* gene having minimal or no detectable CIS activity. Furthermore, high-temperature processing (fried chips) of transgenic derived tubers resulted in light brown products having a significantly lowered level of dietary acrylamide. Importantly, in downstream characterization, few of transgenic lines showed a complete absence of TALENs sequences, thus offering a transgene-free approach.

Other efforts to incorporate nutritional traits in potato include starch alterations using TALENs (Kusano et al., 2016) and CRISPR/Cas9 (Andersson et al., 2017). Through designing a novel delivery system, termed “Emerald–Gateway TALEN system,” Kusano et al. (2016) targeted a host gene, *Granule-bound starch synthase* (*GBSS*) in potato for site-specific mutation. *GBSS* is predominantly involved in amylose biosynthesis during starch granulation. Its disruption may reduce the amylose content and amylose/amylopectin ratio (Zeeman et al., 2010), and thus might affect starch quality in potato tubers. *Agrobacterium*-mediated transformation of potato cells with TALEN constructs resulted in three types of stable mutations in regenerated lines, dominantly having a deletion mutation (63 nucleotides deletion) (Kusano et al., 2016). Recently, CRISPR/Cas9 technology has been used to efficiently silence the *GBSS* in potato cv. Kuras (Andersson et al., 2017). Protoplast transformation with CRISPR/Cas9 constructs resulted in a site-specific mutation in all (four) alleles of *GBSS* gene in 2% of transformed lines. Full knock-out of targeted genomic sites resulted in complete loss of the *GBSS* activity and yielded an altered starch quality in transgenic tubers when compared to controls (Andersson et al., 2017). Genome-wide analyses coupled with transcriptomics, proteomics and metabolomics will further dissect the molecular basis of starch-related traits of potato and could facilitate/accelerate the starch modifications by using NBTs. The production of high-quality starch in potato may be of current research interest to meet the demands of food and industrial sectors.

In another study, Butler et al. (2016) utilized a geminivirus replicon (GVR) vector for delivering sequence-specific nucleases (SSNs) to target the potato herbicide tolerance gene *ALS1* and regenerated transformants carrying a point mutation in *ALS1* gene were confirmed for herbicide susceptibility. Forsyth et al. (2016) reported the targeted integration of transgene into a pre-selective, transcriptionally active site of potato genome using TALEN system coupled with a molecular marker, i.e., the mutated *Acetolactate synthase* (*ALS*) gene. Potato *Ubi7* (constitutively expressing gene) was selected as a target for TALEN and after its functional confirmation in a transient system (*N. benthamiana*), *Agrobacterium*-mediated transformation was used to develop transgenic potatoes (*S. tuberosum* cv. Ranger Russet) (Forsyth et al., 2016). Importantly, the molecular confirmation of transgenic lines showed a single copy of transgene in most of the regenerated events. This could help in downstream transgenic characterization by reducing the workload of generating multiple independent lines for random transgene insertions. Their work established the efficacy of TALENs for achieving a more precise and site-specific genome editing in potato for trait incorporation. However, stable transformation of TALEN reagents carrying bacterial genes (TALE DNA binding domain from Xanthomonas) in plants may trigger the GM concerns having transgenes as codified by the regulatory authorities.

**Figure 1:**
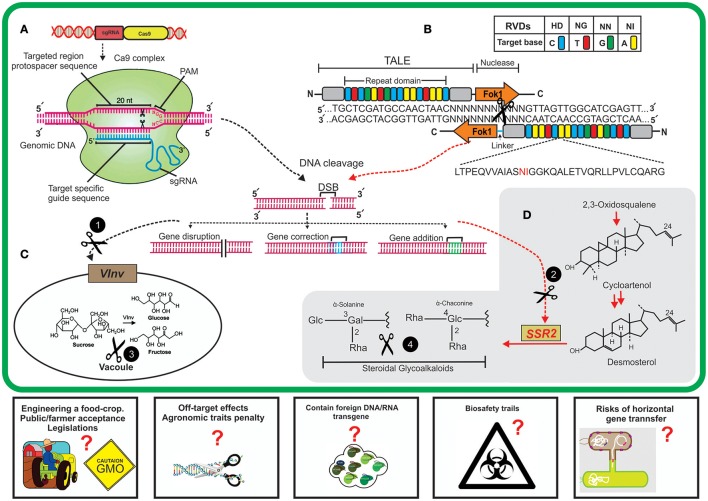
A schematic diagram of new breeding technologies (NBTs) application for editing potato genome for nutritional improvement. **(A)** Clustered regularly interspaced short palindromic repeat/CRISPR associated9 (CRISPR/Cas9) system. Expression of constructs containing a single guide RNA (sgRNA) and Cas9 endonuclease will result in the assembly of sgRNAs and Cas9 nuclease to make a sgRNA/Cas9 complex. The designed sgRNA having sequence complementarity will bind specifically to a targeted site on genomic DNA and sgRNA/Cas9 complex will cleave 3' upstream of PAM (protospacer adjacent motif) sequence; shown by black scissors. This cleavage will result in double-stranded brakes (DSB) in targeted genome. **(B)** Transcription activator-like effector nucleases (TALENs) system. The TALE array contains a highly conserved (33–34 nt) DNA binding domain having repeat variable di-residues (RVDs) at positions 12 and 13 to guide the target-specific binding. Nuclease activity is performed by domains containing *FokI* endonucleases to produce DSBs. These DSBs are normally repaired by host-mediated DNA repair mechanisms which might results in targeted mutation and end in either gene disruption, correction or addition. The black circles having white text (1,2) represent the CRISPR/Cas9/TALENs cleavage of two host genes (vacuolar invertase, *VInv*; sterol side chain reductase, *SSR2*). **(C)** *VInv* is primarily involved in bioconversions of sucrose to fructose and glucose inside cell vacuole, precursors of acrylamide formation. **(D)** Biosynthesis of steroidal glycoalkaloids in plant cell from cycloartenol which is mediated by the activity of host *SSR2* gene. NBTs-mediated targeting of host genes will result in reduced formation of anti-nutrients (acrylamide and steroidal glycoalkaloids) inside tubers and thus result in the improved quality of potato tubers. The proposed challenges (rectangles) by using these technologies might result in some questions such as society and regulation regimes' approval for editing food crop, off-site targeting effects on plants, the presence of any transgene, biosafety trails to check health-related issues, and the potential risks of horizontal gene transfer by using these GM crops. These questions need to be addressed while before using some NBTs.

Recently, transient expression of TALENs, delivered through non-viral *Agrobacterium-mediated* transformation, yielded targeted mutations in two potato cultivars, Russet Burbank and Shepody (Ma J. et al., 2017). The infiltrated TALEN constructs were meant to induce mutations in two different host genes, i.e., (i) *1,4-alpha-glucan branching enzyme* (*SBE1*), (ii) *Vacuolar invertase 2* gene (St*vacINV2*). The regenerated lines were confirmed for targeted chromosomal mutation through deep sequencing (Illumina), that revealed three types of induced mutations having dominantly deletion mutations in both of cultivars. TALEN technology through agroinfiltrations could be effectively used to induce targeted mutation for improving some elite potato cultivars (Ma J. et al., 2017). Figure 1 illustrates a schematic model of NBTs application for incorporating desired modifications in the potato genome to enhance nutritional improvements.

ZFNs are first-generation genome editing nucleases engineered to make DSBs through fusions of artificial, sequence-specific zinc finger proteins with the nonspecific DNA cleavage domains of the *Fok*I restriction endonuclease (Kim and Kim, 2014). The applications of ZFNs for genetic engineering has been limited to crops like tobacco (Cai et al., 2009), *Arabidopsis* (Zhang et al., 2010), and soybean (Curtin et al., 2011) and not in potato and other horticultural crops (Gaur et al., 2018). The limited examples of ZFNs-mediated genome modification in plants are might be due to some disadvantageous such as low success rate (~24%), low or variable mutation rate (~10%), high off-target effects, and technically difficult in designing feasibility (Xiong et al., 2015; Zhang H. et al., 2017). These challenges have greatly narrowed the spectrum of ZFNs technology for adoption by the scientific community.

The studies discussed in this section provide comprehensive information regarding the utility of some NBTs for potato genome editing. Although, the applications of these tools are unlimited in the context of genetic engineering, the selection of suitable genomic targets and efficient editing tool is a critical prerequisite to get the desired goals. For example, transient expression of TALEN/CRISPR system could incorporate desired traits without stable integration of the transgene. Edited crops having non-detectable foreign DNA/RNA could face less opposition in regulatory/public clearance and could seem in line with their natural variants. NBTs could offer promising solutions to engineer complex genomic traits involving several molecular pathways like synthesis of starch, proteins, vitamins, etc., that otherwise would require tedious multistep engineering using conventional techniques. Other application may include functional studies of uncharacterized genes in potato using NBTs that could facilitate more precise and site-specific mutation at targeted loci. Furthermore, the induced mutations could be mapped by utilizing various next-generation sequencing (NGS) techniques. This could save the time for estimating off-site targeting effects that may or may not phenotypically appear later during crop growth. Conclusively, NBTs could be effectively used to engineer a number of nutritional traits in potato like enhanced protein content (Chakraborty et al., 2010), vitamin C content (Bulley et al., 2012), β-carotene level (Li et al., 2012), and others etc.

## Gm potato commercialized so far: risk assessments and regulations

The expansion of biotech crops over last two decades has firmly established the role of genetic engineering in modulating various agronomical, environmental and predominantly health-related traits in plants. Today, more than two billion hectares of agricultural land is under cultivation of biotech crops, which signifies its importance and adaptability to meet future challenges through generating useful phenotypes in plants (Parisi et al., 2016). Most of the GM potato cultivars commercialized so far include trait incorporations such as resistance to viruses and other phytopathogens (Ricroch and Henard-Damave, 2016). The first GM potato appeared in the market in 1995 was named “NewLeaf” by Monsanto®, which was genetically engineered using a toxin *Bt* gene to generate resistance against Colorado beetle (*Leptinotarsa decemlineata*) (Kilman, 2001). Another engineered potato variety appeared in March 2010; a GM potato “Amflora,” developed by BASF Plant Science and aimed at improved amylopectin content (waxy tuberous starch) for the processing industry, was approved by the European Commission (Lucht, 2015; Zaheer and Akhtar, 2016). A total of 23 GM potato lines are in the regulatory approval process (10 at precommercial, 11 at regulatory, and 2 at advance development stages) that has been engineered for various agronomical and quality related traits (Parisi et al., 2016). In 2017, the U.S. Environmental Protection Agency (EPA) and the Food and Drug Administration (FDA) has approved the cultivation of three GM potatoes (Innate^TM^ Second-Generation; developed by Simplot corp.®) meant to resist fungal (late blight) infections, and acrylamide formation (https://durangoherald.com/articles/140336-u-s-approves-3-types-of-genetically-engineered-potatoes?wallit_nosession=1). Importantly, these GM potatoes were developed using various modern breeding tools and got regulatory approvals. A number of other potato varieties engineered for various agronomic traits are in the pipeline or subjected to biosafety and field trials in different countries (Ricroch and Henard-Damave, 2016; Table 3).

The development and commercialization of GM crops is a huge challenge for scientists and regulatory domains due to multiple technical, ethical and social/public limitations (Podevin et al., 2012). In addition, the socioeconomic benefit of utilizing these GM products is a big constraint to their producer and consumer adaptations. Still, the costs related to GM development/approval usually exceed $35 million (Smyth et al., 2017), which seemed to be unsuitable for many low-income public-private institutions. This limits the interest of investing in GM technology in many developing countries (Pérez-Massot et al., 2013). The public acceptance in adopting GM-labeled products further makes it questionable for legislation, government and/or environmental authorities. With technological advancement, the NBTs could be used to generate a highly specific genetic modification that is indistinguishable from natural variants/mutants and therefore significantly reduce the GM concerns (Wolt et al., 2016). For example, Cellectis® (a multinational biotech company) developed a transgene-free potato in 2014 engineered for improved processing traits (Wolt et al., 2016). They used TALEN technology to introduce a base deletion in potato genome through protoplast transformation of an exogenous genetic material from some plant pest (*Phytophthora infestans*). Phenotypic and molecular confirmation of regenerated products showed no detection of any exogenous material in segregating generation, thus, being transgene-free, APHIS (a USDA regulatory domain under the Plant Protection Act: CFR 7) did not take it under regulatory process (Wolt et al., 2016).

## Conclusion and future prospects

Research focusing on food safety and security can provide substantial ways to meet up the rising food demands, especially in the food-deficit countries. The rapid development of plant genetic engineering has provided new exciting tools to generate crops with enhanced yield and nutritional traits. In this context, potato crop has enormous potential to contribute to food security as it could provide low-cost, high energy food at sustainable basis (Zaheer and Akhtar, 2016). Several studies have demonstrated the incorporation of nutritional traits in potato such as enhanced protein content (Chakraborty et al., 2010), vitamin C content (Bulley et al., 2012), β-carotene level (Li et al., 2012), triacylglycerol (Hofvander et al., 2016), tuber methionine (Kumar and Jander, 2017), and amylose content (Krunic et al., 2018; Table 1). Other research priorities are given to reduce anti-nutritional compounds in potatoes such as steroidal glycoalkaloids (Itkin et al., 2013), acrylamide (Clasen et al., 2016) and other food toxins (Hajeb et al., 2014; Table 1). Recently, the emergence of NBTs such as TALENs, ZFNs, CRISPR/Cas9 etc. has provided opportunities for a robust, precise, and site-specific genome editing to introduce important agronomical traits in various crop plants (Mahfouz et al., 2016; Weeks, 2017).

Within the context of potato genome editing, ongoing research is focused on utilizing NBTs to incorporate important traits (Table 2). However, most of these efforts generated end products having transgenic tags, being questioned by food safety, legislation, and extensive consumer opposition. To circumvent these regulatory barriers, NBTs research should now focus on generating transgene-free products, specifically in case of food crops (Wolt et al., 2016). Since, in vegetatively propagated crops like potato, the procedure for transgene removal in subsequent generations through segregation is time-consuming, the utilization of agroinfiltration and protoplast transformation to deliver NBTs' reagents provide a rational procedure for transgene-free potato production (Bortesi and Fischer, 2015). The CRISPR/Cas9 approach can be utilized to incorporate nutritional improvement in potato coupled with late blight resistance through transient expression of transcription factor (*StWRKY1*) in a transgene-free method (Yogendra et al., 2015). Other research priorities could focus on eliminating allergen compounds in potato such as alkaloids, glycoprotein patatin etc. (Zaheer and Akhtar, 2016). In addition, incorporation of abiotic (environmental, salinity, drought, temperature) stress resistance coupled with increased nutrition could facilitate potato to acclimatize in diverse agro-ecological zones, thus impeding food-shortage in less fertile/water deficit agricultural lands. The introduction of pest resistance into commercial cultivars would reduce the pesticide applications, thus impeding the environmental pollution. Further expansion of nutritional studies can set some preliminary values to justify the health benefits of potato-derived foods. Research efforts are needed to mitigate the mechanisms of nutrient-loss, such as co-pigmentation and to enhance the health-promoting components such as antioxidants and phytochemicals in commercial cultivars of potato.

The availability of potato genome sequence (www.potatogenome.net) has facilitated the comparative genomic analyses to identify the genes useful for improving several agronomically important traits like tuberization, loss of bitterness, and diseases resistance (Hardigan et al., 2017; Li et al., 2018). The NBTs offer fast-track development of commercial potato cultivars such as Russet Burbank, Désirée, Kathadin etc. with superior traits such as improved nutrition, biotic and abiotic stress tolerance, and enhanced yield. However, to achieve such goals, it is paramount to acknowledge that not a single GE approach is sufficient to incorporate all the desired traits, rather an integration of NBTs coupled with well-established conventional breeding techniques will be needed. Here, we believe that the future of GM potato is reliant not only on some consumer-oriented traits such as fortified nutrition, enhanced flavor and appearance, but also on some industrial traits such as enhanced starch quality, and reduced CIS activity, which will ultimately enhance the marketability and long-term acceptability of GM potato.

## Author contributions

SM provided the outlines of the review and contributed the key ideas. AH, SS and SZ wrote the manuscript and prepared the figures. SM, AH, and SZ worked on and improved the original draft and figures. The manuscript was approved by all co-authors.

### Conflict of interest statement

The authors declare that the research was conducted in the absence of any commercial or financial relationships that could be construed as a potential conflict of interest.
